# Supplementary material for: A synergistic workspace for human consciousness revealed by Integrated Information Decomposition
Source: eLife. 2024 Jul 18;12:RP88173. doi: 10.7554/eLife.88173 (PMC11257694; doi:10.7554/eLife.88173)
Supplement: MDAR checklist [file elife-88173-mdarchecklist1.docx]

| **Newly created materials** | **Indicate where provided: section/figure legend** | **N/A** |
| --- | --- | --- |
| The manuscript includes a dedicated "materials availability statement" providing transparent disclosure about availability of newly created materials including details on how materials can be accessed and describing any restrictions on access. | Data Availability section |  |
| **Antibodies** | **Indicate where provided: section/figure legend** | **N/A** |
| For commercial reagents, provide supplier name, catalogue number and RRID, if available. |  | N/A |
| **DNA and RNA sequences** | **Indicate where provided: section/figure legend** | **N/A** |
| Short novel DNA or RNA including primers, probes: Sequences should be included or deposited in a public repository. |  | N/A |
| **Cell materials** | **Indicate where provided: section/figure legend** | **N/A** |
| Cell lines: Provide species information, strain. Provide accession number in repository OR supplier name, catalog number, clone number, OR RRID. |  | N/A |
| Primary cultures: Provide species, strain, sex of origin, genetic modification status. |  | N/A |
| **Experimental animals** | **Indicate where provided: section/figure legend** | **N/A** |
| Laboratory animals or Model organisms: Provide species, strain, sex, age, genetic modification status. Provide accession number in repository OR supplier name, catalog number, clone number, OR RRID. |  | N/A |
| Animal observed in or captured from the field: Provide species, sex, and age where possible. |  | N/A |
| **Plants and microbes** | **Indicate where provided: section/figure legend** | **N/A** |
| Plants: provide species and strain, ecotype and cultivar where relevant, unique accession number if available, and source (including location for collected wild specimens). |  | N/A |
| Microbes: provide species and strain, unique accession number if available, and source. |  | N/A |
| **Human research participants** | **Indicate where provided: section/figure legend** | **N/A** |
| If collected and within the bounds of privacy constraints report on age, sex, gender and ethnicity for all study participants | Methods section; subsections “Anaesthesia Data: Recruitment” and “Disorders of Consciousness Patient Data: Recruitment”.  Healthy volunteers (n=19) were recruited (18–40 years; 13 males). Volunteers were right-handed, native English speakers, and had no history of neurological disorders. In accordance with relevant ethical guidelines, each volunteer provided written informed consent, and received monetary compensation for their time.  A total of 71 DOC patients were recruited from specialised long-term care centres from January 2010 to December 2015. Of the initial sample of 71 patients who had been recruited, a total of 22 adults (14 males; 17–70 years; mean time post injury: 13 months) meeting diagnostic criteria for Unresponsive Wakefulness Syndrome/Vegetative State or Minimally Conscious State due to brain injury were included in this study. |  |
| **Study protocol** | **Indicate where provided: section/figure legend** | **N/A** |
| If the study protocol has been pre-registered, provide DOI. For clinical trials, provide the trial registration number OR cite DOI. |  | N/A |
| **Laboratory protocol** | **Indicate where provided: section/figure legend** | **N/A** |
| Provide DOI OR other citation details if detailed step-by-step protocols are available. |  | N/A |
| **For in vivo studies: State whether and how the following have been done** | **Indicate where provided: section/figure legend** | **N/A** |
| Sample size determination | Not done (reuse of previously collected datasets) |  |
| Randomisation |  | N/A |
| Blinding | Since the patients' status as DOC patients was evident, no researcher blinding was possible. For the same reason, anaesthetised status is evident and blinding was not possible. |  |
| Inclusion/exclusion criteria | Methods section; subsections “Anaesthesia Data: Recruitment” and “Disorders of Consciousness Patient Data: Recruitment” Anaesthesia: Due to equipment malfunction or physiological impediments to anaesthesia in the scanner, data from n=3 participants (1 male) were excluded from analyses, leaving a total n=16 for analysis  DOC patients: before analysis took place, patients were systematically excluded if an expert neuroanatomist blinded to diagnosis judged that they displayed excessive focal brain damage (over one third of one hemisphere), or if brain damage led to suboptimal segmentation and normalisation, or due to excessive head motion in the MRI scanner (exceeding 3mm translation or 3 degrees rotation). |  |
| **Sample definition and in-laboratory replication** | **Indicate where provided: section/figure legend** | **N/A** |
| State number of times the experiment was replicated in the laboratory. |  | N/A |
| Define whether data describe technical or biological replicates. |  | N/A |
| **Ethics** | **Indicate where provided: section/figure legend** | **N/A** |
| Studies involving human participants: State details of authority granting ethics approval (IRB or equivalent committee(s), provide reference number for approval | Methods section; subsections “Anaesthesia Data: Recruitment” and “Disorders of Consciousness Patient Data: Recruitment” Anaesthesia data: The study received ethical approval from the Health Sciences Research Ethics Board and Psychology Research Ethics Board of Western University (Ontario, Canada).  DOC data: Ethical approval for this study was provided by the National Research Ethics Service (National Health Service, UK; LREC reference 99/391). |  |
| Studies involving experimental animals: State details of authority granting ethics approval (IRB or equivalent committee(s), provide reference number for approval |  | N/A |
| Studies involving specimen and field samples: State if relevant permits obtained, provide details of authority approving study; if none were required, explain why. |  | N/A |
| **Dual Use Research of Concern (DURC)** | **Indicate where provided: section/figure legend** | **N/A** |
| If study is subject to dual use research of concern regulations, state the authority granting approval and reference number for the regulatory approval. |  | N/A |
| **Attrition** | **Indicate where provided: section/figure legend** | **N/A** |
| Describe whether exclusion criteria were pre-established. Report if sample or data points were omitted from analysis. If yes, report if this was due to attrition or intentional exclusion and provide justification. | Methods section; subsections “Anaesthesia Data: Recruitment” and “Disorders of Consciousness Patient Data: Recruitment” Anaesthesia: Due to equipment malfunction or physiological impediments to anaesthesia in the scanner, data from n=3 participants (1 male) were excluded from analyses, leaving a total n=16 for analysis  DOC patients: before analysis took place, patients were systematically excluded if an expert neuroanatomist blinded to diagnosis judged that they displayed excessive focal brain damage (over one third of one hemisphere), or if brain damage led to suboptimal segmentation and normalisation, or due to excessive head motion in the MRI scanner (exceeding 3mm translation or 3 degrees rotation). |  |
| **Statistics** | **Indicate where provided: section/figure legend** | **N/A** |
| Describe statistical tests used and justify choice of tests. | Network Based Statistic; Spatial autocorrelation-preserving null model for correlation (reported in section Methods, subsection Statistical Analysis) |  |
| **Data availability** | **Indicate where provided: section/figure legend** | **N/A** |
| For newly created and reused datasets, the manuscript includes a data availability statement that provides details for access (or notes restrictions on access). | The Human Connectome Project datasets are freely available from http://www.humanconnectome.org/. Due to patient privacy concerns, DOC patient data are available upon request by qualified researchers. The UK Health Research Authority mandates that the confidentiality of data is the responsibility of Chief Investigators for the initial studies (in this case, Dr. Allanson and Prof Menon; and anyone to whom this responsibility is handed – for example, in the context of retirement or transfer to another institution). For researchers interested in working with this dataset, please contact Dr. Judith Allanson (judith.allanson@addenbrookes.nhs.uk), Prof. David Menon (dkm13@cam.ac.uk) and/or Dr. Emmanuel Stamatakis (eas46@cam.ac.uk). Requests will be considered on a case-by-case basis, assessing the feasibility and appropriateness of the proposed study, and the capacity to maintain the required levels of data security, consistent with the original approved Research Ethics approval, and the patient information sheet that was the basis of consent obtained. The propofol dataset is available on the OpenNeuro data repository (doi: 10.18112/openneuro.ds003171.v2.0.1). |  |
| When newly created datasets are publicly available, provide accession number in repository OR DOI and licensing details where available. |  | N/A |
| If reused data is publicly available provide accession number in repository OR DOI, OR URL, OR citation. |  | N/A |
| **Code availability** | **Indicate where provided: section/figure legend** | **N/A** |
| For any computer code/software/mathematical algorithms essential for replicating the main findings of the study, whether newly generated or re-used, the manuscript includes a data availability statement that provides details for access or notes restrictions. | The Java Information Dynamics Toolbox v1.5 is freely available online: (<https://github.com/jlizier/jidt>). The CONN toolbox version 17f is freely available online (<http://www.nitrc.org/projects/conn>). The Brain Connectivity Toolbox code used for graph-theoretical analyses is freely available online (<https://sites.google.com/site/bctnet/>). The HRF deconvolution toolbox v2.2 is freely available online: (<https://www.nitrc.org/projects/rshrf>). The code for spin-based permutation testing of cortical correlations is freely available at <https://github.com/frantisekvasa/rotate_parcellation>. We have made freely available MATLAB/Octave and Python code to compute measures of Integrated Information Decomposition of timeseries with the Gaussian MMI solver, at <https://github.com/Imperial-MIND-lab/integrated-info-decomp>. |  |
| Where newly generated code is publicly available, provide accession number in repository, OR DOI OR URL and licensing details where available. State any restrictions on code availability or accessibility. |  | N/A |
| If reused code is publicly available provide accession number in repository OR DOI OR URL, OR citation. | The Java Information Dynamics Toolbox v1.5 is freely available online: (<https://github.com/jlizier/jidt>). The CONN toolbox version 17f is freely available online (<http://www.nitrc.org/projects/conn>). The Brain Connectivity Toolbox code used for graph-theoretical analyses is freely available online (<https://sites.google.com/site/bctnet/>). The HRF deconvolution toolbox v2.2 is freely available online: (<https://www.nitrc.org/projects/rshrf>). The code for spin-based permutation testing of cortical correlations is freely available at <https://github.com/frantisekvasa/rotate_parcellation>. We have made freely available MATLAB/Octave and Python code to compute measures of Integrated Information Decomposition of timeseries with the Gaussian MMI solver, at <https://github.com/Imperial-MIND-lab/integrated-info-decomp>. |  |
| **Adherence to community standards** | **Indicate where provided: section/figure legend** | **N/A** |
| State if relevant guidelines (e.g., ICMJE, MIBBI, ARRIVE, STRANGE) have been followed, and whether a checklist (e.g., CONSORT, PRISMA, ARRIVE) is provided with the manuscript. |  | N/A |
